# “Knowing It Before Blocking It,” the ABCD of the Peripheral Nerves: Part C (Prevention of Nerve Injuries)

**DOI:** 10.7759/cureus.41847

**Published:** 2023-07-13

**Authors:** Kartik Sonawane, Hrudini Dixit, Kaveri Mehta, Navya Thota, Palanichamy Gurumoorthi

**Affiliations:** 1 Anesthesiology, Ganga Medical Centre and Hospitals, Pvt. Ltd, Coimbatore, IND; 2 Anesthesiology, Sir H. N. Reliance Foundation Hospital and Research Centre, Mumbai, IND; 3 Anesthesia and Critical Care, Corniche Hospital, Abu Dhabi, ARE

**Keywords:** regional anesthesia, safe regional anesthesia, pressure manometers in peripheral nerve blocks, prevention of nerve injuries, nerve injuries

## Abstract

“A clever person solves the problem. A wise person avoids it” (Albert Einstein). There is no convincing evidence that any modality 100% effectively prevents nerve injury. The risk of nerve injury remains the same even with the ultrasound due to limitations in the resolution of images and inter-operator and inter-patient differences. In a nutshell, caution is required when dealing with precious nerves in the perioperative period, either during peripheral nerve blocks (PNBs), patient positioning, or surgery. Identifying pre-existing nerve injury, either due to trauma or an existing neuropathy, and preventing further nerve injury should be an important goal in providing safe regional anesthesia (RA).

Multimodal monitoring is key to avoiding multifactorial nerve injuries. The use of triple guidance (ultrasound + peripheral nerve stimulator + injection pressure monitor) during PNBs further improves the safety of RA. The ultrasound helps in real-time visualization of the nerve, needle, and drug spread; the peripheral nerve stimulator helps confirm the target nerves; and the injection pressure monitor helps avoid nerve injury. Such multimodalities can also give the confidence to administer PNB without risk of nerve injury.

This article is part of the comprehensive overview of the essential understanding of peripheral nerves before blocking them. It describes various preventive measures to avoid peripheral nerve injuries while administering PNBs. It will help readers understand the importance of prevention in each step to avoid perioperative PNIs.

## Introduction and background

Suffering from neurological injuries in the perioperative period is a kind of psychological trauma for the patient. Due to multifactorial origin, nerve injury can occur at any step in the perioperative period, such as during peripheral nerve block (PNB) administration, ongoing surgery, or the postoperative period. Preventing nerve injuries can be possible by eliminating all potential causes and taking precautions at every step rather than just focusing on one aspect. The ultimate goal is to provide safe and effective regional anesthesia (RA). Achieving this requires a basic understanding of the structures, equipment, modalities, and people involved. Modern RA practices facilitate providing targeted RA training, obtaining basic knowledge of the target structures and modern equipment, and creating red flags to review each step to avoid complications.

The advent of ultrasound guidance (USG) in RA and various available objective monitoring techniques to avoid nerve injuries have made RA practice much safer. However, ultrasound has been implicated and proven revolutionary in RA practice, but it fails to accurately locate intrafascicular or extrafascicular needle tips. Hence, the risk of nerve injury remains the same even with the ultrasound due to limitations in the resolution of images and inter-operator and inter-patient differences. Therefore, like multimodal analgesia for multifactorial pain, multimodal monitoring may be useful in preventing peripheral nerve injuries (PNIs) of multifactorial origin.

Multimodal monitoring includes using multiple objective modalities such as ultrasound, peripheral nerve stimulators (PNSs), and injection pressure monitoring techniques. Using ultrasound allows real-time visualization of the needle and local anesthetic (LA) deposition. PNS helps confirm target nerves and avoid intraneuronal injection. Injection pressure monitoring devices are designed to avoid intraneuronal injections by stopping injection at high opening pressures. So far, three injection pressure monitoring devices are commercially available: BSmart™ (by B.Braun Medical, Melsungen, Germany), NerveGuard (by Pajunk Medical Systems, Geisingen, Germany), and SAFer Injection for Regional Anesthesia (SAFIRA™) (by Medovate Ltd, Girton, Cambridge, UK). The NerveGuard and SAFIRA are essentially in-line pressure limiters that stop injections automatically once the pressure reaches above 15-20 pounds per square inch (psi) (>15 psi for the NerveGuard and >20 psi for the SAFIRA). The BSmart offers the advantage of using the decision-making capacity to inject at any indicated pressure. However, no convincing evidence exists that any modality is 100% effective in preventing nerve injury [1-3].

This article is part of the comprehensive overview of the essential understanding of peripheral nerves before blocking them. It describes various preventive measures to avoid PNIs while administering PNBs. It will help readers understand the importance of prevention in each step to avoid perioperative PNIs.

## Review

This narrative overview describes various preventive measures to avoid PNIs while administering PNBs. Related literature searches were performed using online platforms (PubMed, Medline and Embase databases, Cochrane Library, and Google Scholar) using relevant search terms (neurons/nerve damage/nerve injuries/prevention of nerve injuries/monitoring in RA/injection pressure monitors/in-line pressure manometers/recent advances in ultrasound to prevent PNIs). Articles published in English were selected (year 2000-2023), and their reference sections were manually searched for additional information.

Prevention of nerve injuries

**Figure 1:**
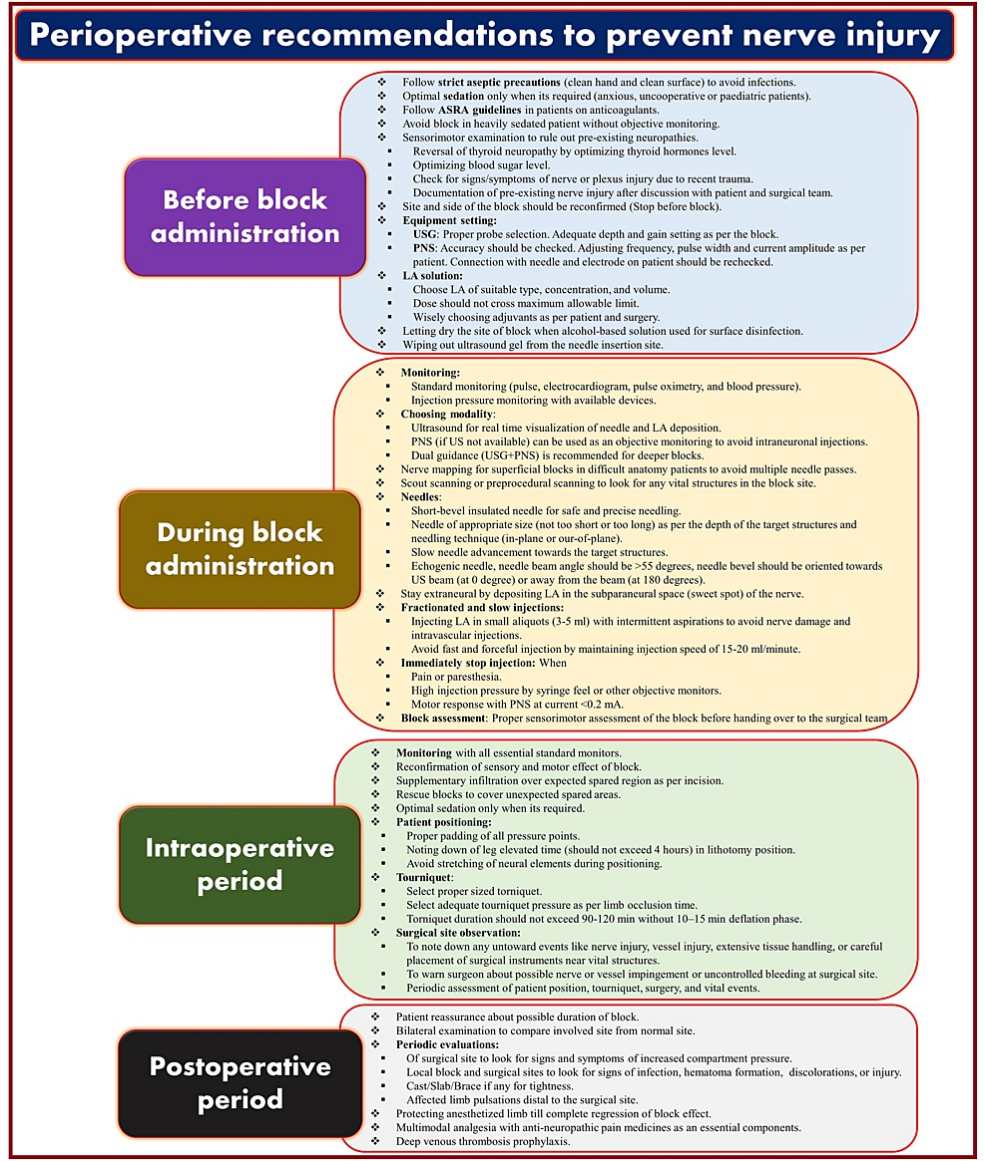
Perioperative recommendations to prevent peripheral nerve injuries. Source: This figure was created by the first author (K.S.). ASRA, American Society of Regional Anesthesia; USG, ultrasound guidance; PNS, peripheral nerve stimulator; LA, local anesthetic; US, ultrasound

Prevention is always better than cure. All preventive measures are very effective if implemented on all fronts (Figure 1), i.e., before and during the block and surgery and in the postoperative period. Before administrating PNB, the evaluation of the patient to screen for risk factors causing nerve injuries and noting existing neuropathy is very important. Patients with such neuropathy may have enough information about it or can glean it from analysis of past clinical records and current medications. In such patients, existing sensory-motor deficits can be reconfirmed by clinical examination, and the details are further explained to the patient as necessary. It should be documented, and written informed consent should also be obtained for possible nerve injury. Drug history can provide insight into the status of neuropathic disorders and help follow coagulation-related guidelines in patients taking antiplatelet drugs or anticoagulants to avoid associated nerve injury.

**Figure 2:**
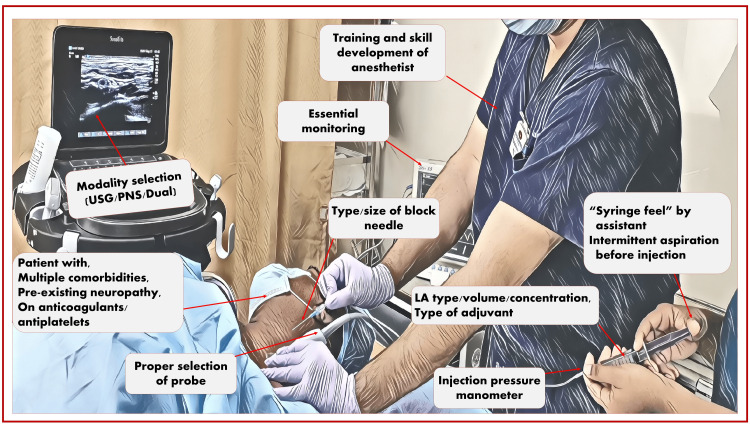
Important factors to prevent nerve injuries during the administration of peripheral nerve block. Source: This figure was created by the first author (K.S.). USG, ultrasound guidance; PNS, peripheral nerve stimulator; LA, local anesthetic

When administering PNB, attention can be paid to many important factors (Figure 2) to prevent nerve injury and ensure safe RA. Evidence shows that the experience and skill of the regional anesthetist can directly impact the overall outcome of RA. Therefore, proper training and skill development play an important role in providing safe RA and thereby preventing PNI. The needle’s type/size/design plays an important role in preventing nerve injury by avoiding or limiting damage to nerve structure or by facilitating the visibility of the needle tip on ultrasound. LAs and adjuvants for preventing nerve injuries also play an important role. High-concentrated LAs are considered to be more neurotoxic than low-concentrated LAs [4-6]. Therefore, selecting LA type/concentration/volume and adjuvant depending on the purpose (anesthesia or analgesia) must be carefully decided before planning an appropriate RA technique.

The available assistant should know the importance of intermittent aspiration during the injection and avoid high-pressure injections, felt by the tactile feel of the syringe. Various syringes and in-line connectors used during PNB can also play an important role in preventing nerve injury. The modalities such as ultrasound, PNS, and dual guidance (USG + PNS) offer their advantages in preventing nerve injuries. Most important is the monitoring during the performance of PNB. Such monitoring can be subjective, relying entirely on the subject (patient) and their symptoms (such as paresthesia/pain) or on the assistant’s tactile sense of high injection pressure while injecting the drug. Objective monitoring helps prevent intraneuronal injection by either automatically stopping the injection at high pressure or displaying the pressure at injection, prompting the anesthetist to decide on the injection. After PNB, all important precautions should be followed during the perioperative period. Patient follow-up is very important to identify, evaluate, diagnose, or explain neurological deficits, and to reassure the patient about them.

Structured RA training

Most PNB-related nerve injuries are related to needle trauma or needling technique. Acquiring needling skills during RA training is important to ensure safe RA without causing nerve injury. The entire success of ultrasound-guided RA (UGRA) depends on a triad of three distinct but interrelated skills: image acquisition, correct interpretation of the sonoanatomy, and hand-eye coordination to visualize the needle in real-time while reaching the target nerves. The needle visualization is a dynamic process that often requires constant needle manipulation with simultaneous adjustments of the ultrasound transducer. There can be an extensive learning curve when acquiring UGRA skills [7]. Proper training in this upcoming field is essential, focusing on the technical, non-technical, and scientific aspects. Such training leads to the upgradation of anatomical knowledge, fine motor skills while doing needling, basic knowledge of the knobology of the machine, and identification and management of complications. Traditionally, such training is given by directly observing the expert in the field and performing basic blocks directly on patients under the supervision of an expert.

Needling in a living patient by inexperienced hands harbors a high potential for injury to nerves, vessels, or other vital structures. To overcome this hurdle, looking for alternative training tools that provide at least basic training in a controlled environment is important. Simulation training is one option where different scenarios can be created, and various techniques or strategies can be taught effectively without hurting any live patient. Such training was used as early as 1964 to test the acquisition of cardiopulmonary resuscitation skills with a Resusci-Anne mannequin in paramedics and laypersons [8]. Such simulation training before handling patients can directly help increase patient safety, patient acceptance of the RA technique, and accuracy. After such training, the trainees can be well versed with basic anatomy and ultrasound machines and, most importantly, needling techniques under ultrasound. A simulation-based UGRA curriculum can enhance the learning of anatomy and sonoanatomy, facilitate acquiring UGRA technical and non-technical skills, and provide recurrent training outside the clinical setting. It helps trainees obtain basic skills and perform various RA techniques on live patients without committing gross mistakes and hurting patients much due to reduced attempts.

Monitoring modalities during RA

**Figure 3:**
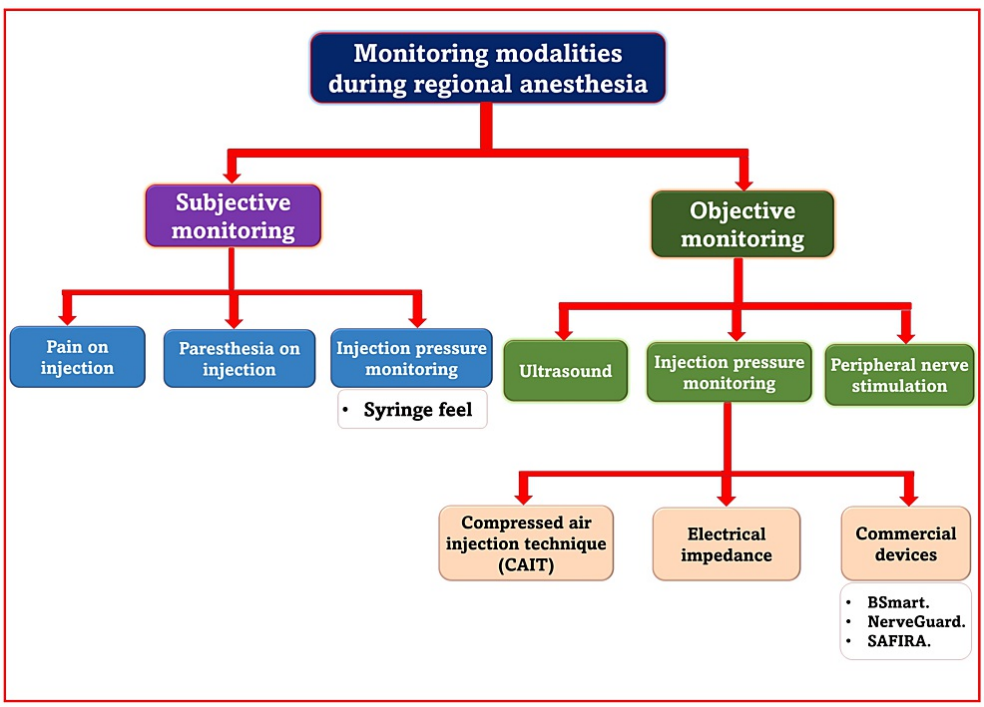
Monitoring modalities during regional anesthesia Source: This figure was created by the first author (K.S.). SAFIRA, SAFer Injection for Regional Anesthesia

Avoiding nerve injuries from various sources (needle, drug, tourniquet, pressure points, positioning during surgery, or surgery itself) can be possible by anticipating such damages before their occurrence and adopting preventive and safety measures. Several techniques have been advocated to improve safety during the execution of PNBs that facilitate vigilant monitoring to avoid nerve injuries. Such monitoring can be subjective and objective based on the patient and devices (Figure 3).

Sometimes, PNB is administered to sleeping/heavily sedated/constrained patients such as pediatric, mentally incompetent, traumatically injured, and intubated patients. Sometimes, it is managed as a rescue or repeat block. In such cases, using more objective monitors than the patient’s subjective symptoms can provide greater confidence in avoiding nerve injury. Therefore, more attention should be paid to objective rather than subjective monitoring. Subjective monitoring includes paresthesia, pain on injection, or resistance during injection (syringe feel) [9]. It is unreliable since it depends on patient feedback that can vary from person to person. Objective monitoring includes injection port pressure display, PNS, compressed air injection technique (CAIT), real-time visualization under ultrasound, and injection pressure manometers such as BSmart, NerveGuard, and SAFIRA.

Paresthesia on injection

Paresthesia refers to an abnormal painless skin sensation (burning, pricking, tingling, numbness, or itching) distal to the block site. During PNB, the patient usually experiences electric shock-type paresthesia. Paresthesia is abnormal electrical activity in the nerve caused by nerve irritation due to the pressure of the needle or the injected solution. Therefore, it should always be interpreted as a warning sign of needle-nerve proximity [10-12]. It has only a 38% likelihood of detecting nerve-to-needle contact and cannot be relied upon to signify intraneural placement [13]. According to the available literature, it has a very low sensitivity and specificity as its association with postoperative nerve injury is contradictory [11]. However, persistent paresthesia (suggesting intraneuronal injection) is a common factor in reports of nerve damage. Again, it is a subjective feeling that can differ from person to person and therefore remains an unreliable indicator of intraneuronal injection.

Pain on injection

Intraneural placement of the injection can be exquisitely painful. Since pain is a subjective feeling, which may vary from person to person, assessing pain in terms of intensity and quality is difficult. It is sometimes difficult to differentiate between a common discomfort during LA injections (pressure paresthesia) and pain during intrafascicular injection. Various patient conditions (such as diabetes mellitus), peripheral neuropathy, premedication, and sedation can impair pain perception. There appears to be little evidence that pain on injection is sensitive or specific. Since a fraction of a milliliter is enough to cause irreversible fascicular damage, the patient’s subjective symptoms may be too late [14,15].

Subjective “syringe feel”

While administering ultrasound-guided PNB, the anesthetist mainly focuses on handling the probe and needle while observing the ultrasound image. At the same time, another anesthetist/assistant performs the actual injection. In such a scenario, the assistant feels the actual resistance upon high opening pressure via tactile feedback, which is again prone to subjective bias. If the assistant does not stop injecting upon resistance, it can lead to neuronal damage [16]. Such significant variability among experienced clinicians in perceiving “normal” injection pressures make this subjective method inaccurate and inconsistent [17]. Because small-volume syringes unintentionally generate high injection pressures, using large-volume syringes (20 mL) is recommended for PNB as they enhance tactile feedback when a greater force is required to inject [11].

Electrical nerve stimulation

Peripheral nerve stimulation is based on the relationship between the current required to elicit a motor response and the distance between the needle tip and the nerve [18]. A threshold current of 0.2-0.5 mA has been considered optimal to elicit a motor response without damaging the nerve [19]. Neurostimulation is highly specific for identifying needle tip placement. A motor response at ≤0.2 mA confirms intraneural needle tip location [20]. A stimulation current of >0.5 mA determines the needle tip position away from the nerve. High current levels may still be required to elicit a response even when the needle tip is intraneural [18,21]. Therefore, PNS may not have high sensitivity to detect intraneural needle tip location.

Electrical impedance

PNS used for PNB are constant current generators. It means that despite the changing resistance offered by different tissues in the body, the same current can be generated. Such resistance to the flow of alternating current in an electrical circuit is known as electrical impedance. It depends on the water-lipid ratio of the tissues. Nerves have a higher water-to-lipid ratio than skin, muscle, fat, or bone [22]. Because of this, nerves have greater electrical impedance than the surrounding muscle and interstitial fluid.

The course of the needle from the extraneural to the intraneural compartment can cause a sudden increase in electrical impedance to almost double the value. Therefore, electrical impedance monitoring can detect the placement of the intraneural needle tip. However, the needle tip must be in the nerve for this to work. Such an electrical impedance measurement can also distinguish between the intravascular and intraneural placement of the needle. Tsui et al. found a near doubling of baseline electrical impedance upon intraneural insertion of the needle [22]. However, the increase in electrical impedance upon intravascular placement of the needle was only 2-3 kilohms [22]. Patirick et al. also found that a rise in electrical impedance greater than 4.3% could indicate accidental nerve puncture during PNB [23].

Ultrasonography

The modernization of RA has paved the way for the resurgence of UGRA techniques. Modern UGRA techniques provide a real-time visual representation of the internal anatomy and help increase the nerve block success rate, accelerate the onset time, and reduce the volume and risk of complications of LA [24,25]. USG is now considered the standard of care for PNBs [26] as it offers patients a life-changing experience through a pain-free hospital stay and ambulation. It is also an attractive means of preventing intraneural injection due to real-time imaging of the needle, needle tip, injectate, and the target nerve. The advantages of using ultrasound over PNS include increased speed of onset, fewer needle passes, increased patient comfort, and lower required LA volumes [24].

The advent of USG for nerve blocks has likely led to an increase in recognition of inadvertent intraneural injections, as evidenced by nerve swelling. However, it may not be an essentially effective means of preventing nerve injury. The reliability of ultrasound in keeping the needle tip extraneural depends largely on the skill of the operator and the imaging properties of the needle and tissue. Despite USG, several case reports of accidental nerve (and vascular) punctures highlight that ultrasound monitoring is not a fail-safe method of preventing neurological (and other mechanical) complications [27-29].

**Figure 4:**
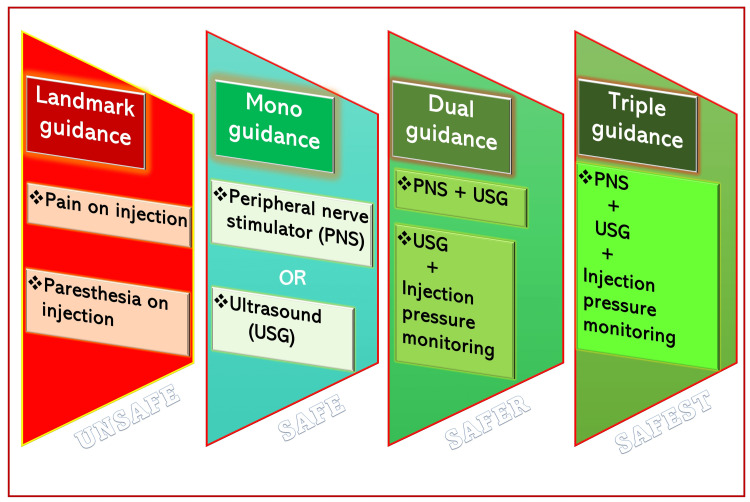
Safety of regional anesthesia as per mono, dual, and triple guidance systems. Source: This figure was created by the first author (K.S.).

**Figure 5:**
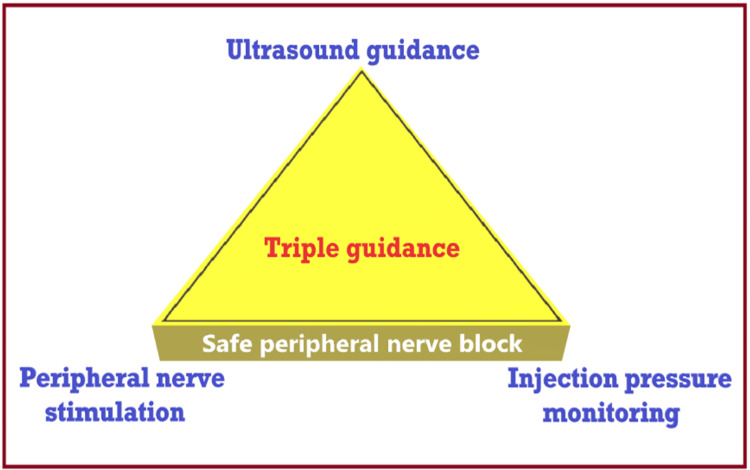
Triple guidance for safe peripheral nerve block. Source: This figure was created by the first author (K.S.).

Furthermore, ultrasound resolution sometimes fails to accurately locate the intrafascicular or extrafascicular needle tip, which is the critical anatomical differentiation required to avoid nerve injury. Finally, by the time the nerve swells in the image, the damage may already have been done by the time the injection is made inside the fascicle. In order to avoid nerve injury, a hydro-dissection technique is recommended while using USG, in which small aliquots of LA are injected during the advancement of the needle toward the target nerve. Dual guidance (USG + PNS) helps reduce the risk of nerve injury by depositing LA around the confirmed nerve (by PNS response) and reducing effective LA volume. In addition, triple guidance (Figures 4, 5) (USG + PNS + injection pressure monitoring) further improves the safety of RA [30,31] and gives the confidence to administer PNBs without nerve injury.

Three-Dimensional and four-Dimensional USG

The conventional two-dimensional (2D) ultrasound offers planar images that require skill and frequent probe maneuvering to optimize the images generated and real-time needle tracking. Various technologies such as three-dimensional (3D) and four-dimensional (4D) USG have emerged in pursuit of better image optimization, real-time needle tracking, and visualization of drug spread. The 3D USG offers the advantage of providing the third volume dimension by providing a 360-degree dataset of the traditional 2D image. It helps get an overview of the spread of the LA around the nerve. Although 3D-USG provides additional volumetric information, it does not help in real-time needle visualization [32,33].

On the other hand, 4D USG can be called real-time 3D USG. It provides a multi-planar view of the sonographic image, aiding in needle tracking, catheter placement, LA spread, and visualization of adjacent structures without extensive probe movement [34]. Currently, the use of 3D and 4D USG is limited by aspects such as the use of expensive software, the use of more computer processing units, the need for specialized probes, heavy probes, limitation of the high-resolution, the portability of USG machines, operator skills, and expertise. Using such technologies in conjunction with echogenic needles and electromagnetic needle tracking can reduce the likelihood of nerve injury.

Artificial Intelligence in Ultrasound

**Figure 6:**
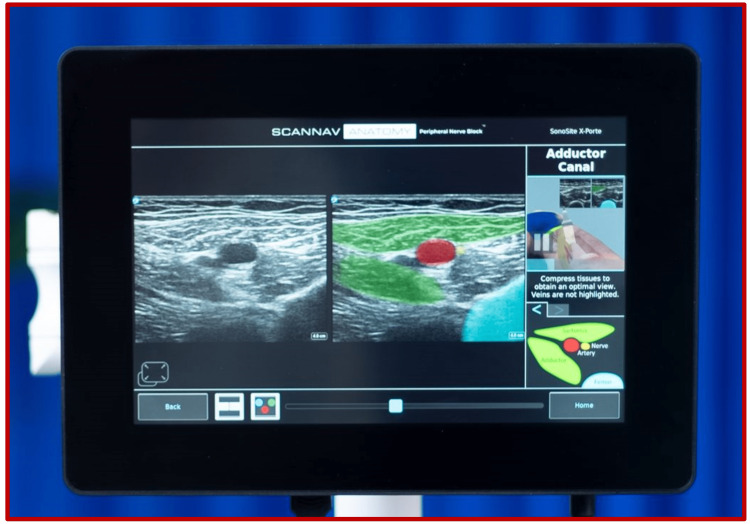
The ScanNav Anatomy Peripheral Nerve Block (Intelligent Ultrasound, Cardiff, UK) technology. Source: https://www.intelligentultrasound.com/wp-content/uploads/2022/10/DSC03437sq-1024x1024.jpg.

The advent of assistive artificial intelligence (AI) has also touched the UGRA sector. The future prospects of this technology are currently being studied, and the possibility of reducing the occurrence of nerve injuries is one area where AI can help find solutions. The ScanNav™ Anatomy Peripheral Nerve Block (Intelligent Ultrasound, Cardiff, UK) is an FDA-approved AI-based technology that generates color overlays and highlights key structures in real-time B-mode USG. It has been trained with more than 800,000 USG images [35,36]. By highlighting and labeling different anatomical structures (Figure 6), AI intelligence can help prevent nerve injuries and other complications, such as inadvertent rupture of vessels, pleura, and peritoneum. Benefits include better image optimization, sonographic image interpretation, real-time needle visualization, needle trajectory guidance, and surrounding structure identification. Therefore, AI will be particularly beneficial in setups with novices and trainees in the UGRA. It can also help reduce the risk of nerve injury and facilitate block success, even for well-trained anesthesiologists and experts in the field.

Injection pressure monitoring

Injection pressure monitoring is useful in detecting needle-nerve contact. It is highly sensitive but lacks specificity. In other words, the absence of high injection pressure effectively rules out an intrafascicular injection [15,37]. High opening injection pressure (>20 psi) determines the intrafascicular placement of the needle tip. Low opening pressure (<20 psi) determines the extrafascicular or extraneural placement of the needle tip. However, high injection pressure can also be caused by needle obstruction, attempted injection into a tendon, or tissue compression caused by the ultrasound transducer.

Despite a lack of paresthesia, PNBs associated with high injection pressure have been reported to result in permanent neurologic injury [38]. As per studies, 40%-70% of injections occur consistently above 20 psi, with a significant portion above 30 psi [21]. Injection at pressures above 20 psi can result in transient nerve damage in up to 8% [39] and serious nerve damage in up to 1% of cases [40]. Therefore, high injection pressure was associated with neurologic deficits and severe axonal damage after the block, in contrast to normal neurological and histological findings with low injection pressure.

**Figure 7:**
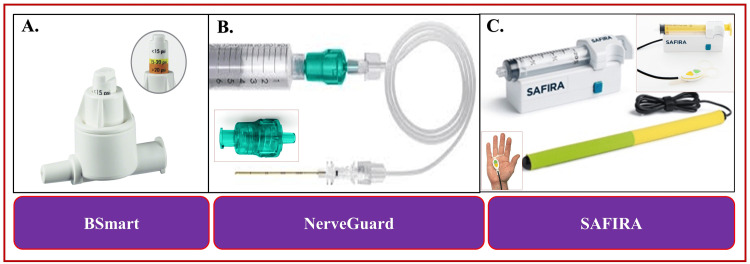
Commercially available in-line pressure manometers. A: BSmart (by B.Braun Medical, Melsungen, Germany). (Source:https://www.bbraun.com/content/dam/catalog/bbraun/bbraunProductCatalog/S/AEM2015/en-01/b5/bsmart.jpeg). B: NerveGuard (by Pajunk Medical Systems, Geisingen, Germany). (Source: https://www.richardsmedical.com/images/Pajunk_NerveGuard_2.jpg; https://pajunk.com/products/regional-anaesthesia/accessories/nerveguard/). C: SAFer Injection for Regional Anesthesia (SAFIRA) (by Medovate Ltd, Girton, Cambridge, UK). (Source: https://www.mercurymed.com/wp-content/uploads/safira1_300x300.jpg https://www.lifescienceindustrynews.com/wp-content/uploads/2021/08/210820_Medovate_NRFit_Hand-Control_8941LR.jpg https://www.mercurymed.com/wp-content/uploads/palm-operator.jpg).

Injection pressure is often assessed using a “syringe-hand-feel” technique, which is prone to subjective bias. Objective injection pressure monitoring is considered safe and effective due to the inability to gauge injection pressure with tactile feedback using the syringe feel technique [9,21]. Objective monitoring of injection pressure can be achieved with the CAIT [41,42] or using commercially available in-line devices (Figure 7) (BSmart, NerveGuard, and SAFIRA).

Compressed air injection technique

**Figure 8:**
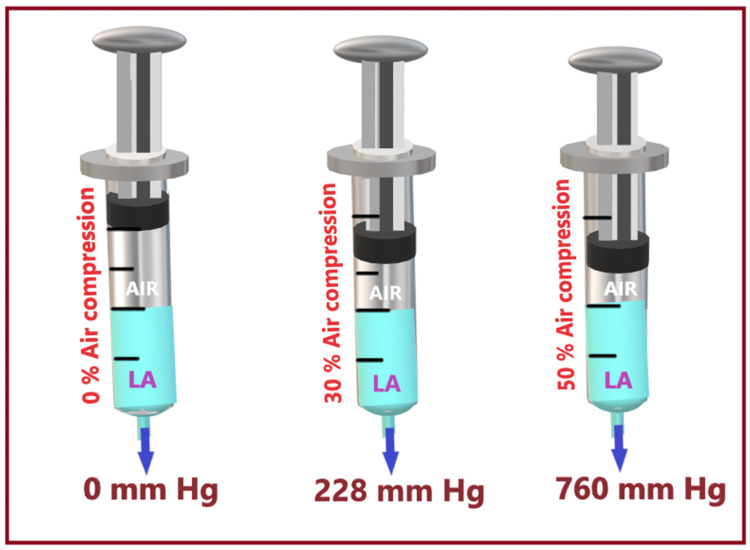
Compressed air injection technique. Source: This figure was created by the first author (K.S.). LA, local anesthetic

This technique is based on Boyle’s law (pressure × volume = constant), i.e., gas or air volume and pressure have an inverse relationship at a constant temperature. It consists of a syringe (20 ml) filled with half a volume of LA (10 ml) and half a volume of air (10 ml) above it (Figure 8). During injection, the air above the LA gets compressed, creating pressure on the LA that is transmitted at the syringe tip. This opening injection pressure can be measured at the syringe tip and maintained at a chosen level by maintaining the air volume compression at a set limit. At 50% air compression, the injection pressure transmitted from the end of the syringe is approximately 1 atm (760 mm Hg) or 14.7 psi, which is within the safe injection pressure threshold, i.e., < 25 psi (1293 mm Hg) [41,42]. Therefore, compressing the air by half its volume (50%) is used as a set or maximum allowable limit below which (<50%) LA can be safely deposited into the extraneural space [15,43]. Thus, this system utilizes compression of a set air volume within the LA syringe to provide real-time objective monitoring and simultaneous control of injection pressure.

In terms of advantages, firstly, it is a simple, more economical, and practical method that constantly keeps the LA injection pressure below the threshold level, thus minimizing the risk of clinically significant nerve injury. Secondly, by decreasing the injection speed, CAIT decreases the risk of intrafascicular injection or siphoning the LA to unwanted tissue planes. Thirdly, the pressures generated by CAIT also remain consistently stable throughout the injection period, unlike the syringe feel technique, which produces high peak pressures. It is likely due to the “cushioning” effect from the volume of air, which dampens the initial high pressure [44].

In terms of disadvantages, firstly, CAIT may also present a potential risk of air injection due to accidental air injection into the vessel, potentially creating a venous air embolism [45]. Secondly, accidental injection of air around the target structures can impair the sonoimage quality due to the hyperechogenicity of the air. Therefore, identifying the neural structures and depositing LA around it can become difficult. Thirdly, the CAIT technique may have potential fluctuations in injection pressure that require accurate aspiration and monitoring of air volumes in the syringe to interpret injection pressure correctly.

BSmart

BSmart is a commercially available easy-to-use in-line injection pressure manometer (Figure 7A) for single-shot and continuous PNB techniques. It facilitates rapid termination of injection upon reaching high pressures. This single-use device is placed in-line between the syringe and injection tubing before the entire system is primed with injectate. It has a color-coded piston, which provides color-coded pressure information by popping up during injection. The white bar on the piston determines <15 psi opening injection pressure, the yellow bar determines <15-20 psi, and the red bar determines pressure >20 psi. The injection pressure depends on the injection speed, the needle size, and the length of the integrated injection tubing [46,47]. High injection pressure (>20 psi) can be due to the location of the needle tip, either intraneural or against bone, tendons, or fascial planes.

Apart from providing enhanced safety of PNBs, the BSmart offers some advantages such as objective monitoring of injection pressure, warning of high injection pressure, prevention of too forceful and rapid injections, consistent monitoring of resistance to injection regardless of who performs the actual injection, and standardized and objective documentation of injection pressure information. However, such assembly may produce false-positive readings during higher injection flow rates [48] due to changed injectate flow characteristics from laminar to turbulent flow during rapid injection or the inherent resistance to flow from the needle shaft [48]. Since this manometer is disposable, it is unknown if its accuracy will degrade with repeated use or if the piston will become less free to move with multiple injections.

NerveGuard

NerveGuard is another in-line automatic injection pressure limiter (Figure 7B), which operates only when the injection pressure is within the limit value (15 psi). If the pressure reaches this threshold value, the NerveGuard valve closes automatically [49,50]. Thus, it avoids nerve damage due to intrafascicular injection by blocking the supply of LA at high injection pressures. It can be easily connected between the syringe and the injection tube. It has a specialized spring mechanism that allows only safe injection opening pressure (<20psi), above which it stops injecting LA solution. No additional features or eye contact (like a colored pop-up piston in BSmart) are required in NerveGuard to avoid high-pressure injections. NerveGuard detects needle/cannula tip occlusion and stops the LA delivery at high injection pressure. This occlusion can be either by a fascia or a needle (cannula-fascia/cannula-nerve contact) in front of the needle/cannula tip. Thus, it gives an important hint for position correction. It is suitable for single-shot or continuous PNBs. NerveGuard offers additional support in locating the tip of the cannula. Together with the automatic pressure limitation, it prevents nerve damage and increases patient safety.

SAFIRA

New technology SAFIRA (Figure 7C) has a built-in safety feature to promote safer injection during RA procedures by preventing injection above 20 psi [51]. Thus, it helps reduce the risk of nerve damage. SAFIRA is designed to use a clinically guided approach to incorporate an engineered safety mechanism that automatically prevents injection above 20 psi. The proprietary syringe works with the driver, calibrated to trigger an alarm upon reaching the maximum injection pressure threshold. It immediately stops further injection automatically, which allows the anesthetist to make immediate checks, such as the position of the needle, and make any necessary adjustments. It cannot be reset to proceed with the injection until the anesthetist agrees, which helps reduce the risk of accidental nerve damage from high-pressure injections.

The traditional practice of RA mainly involves two operators, an anesthetist (to position the needle using an ultrasound probe) and an assistant who injects the LA. In contrast, the SAFIRA system helps single-handedly manage infusion and aspiration while administering RA. It has provided a foot pedal or handheld switch system to control the entire injection. Both the foot pedal and hand switch systems are color-coded (yellow for aspiration and green for injection). The automatic built-in safety feature in SAFIRA stops injection at pressures >20 psi, giving anesthetists more confidence to provide safe RA [52].

## Conclusions

Thus, nerve injuries of multifactorial origins can be prevented by taking necessary undue precautions and using multimodal guidance at each step perioperatively. However, which of many is the best preventive measure has yet to be determined. Further studies and innovations are always welcomed to ensure safe RA.
